# A method for identification of highly conserved elements and evolutionary analysis of superphylum Alveolata

**DOI:** 10.1186/s12859-016-1257-5

**Published:** 2016-09-20

**Authors:** Lev I. Rubanov, Alexandr V. Seliverstov, Oleg A. Zverkov, Vassily A. Lyubetsky

**Affiliations:** Institute for Information Transmission Problems (Kharkevich Institute), Russian Academy of Sciences, Bolshoi Karetnyi per. 19, Building 1, Moscow, 127051 Russia

**Keywords:** Phylogeny, Ultraconserved element, Highly conserved element, Dense subgraph, Apicomplexan parasites, Alveolates

## Abstract

**Background:**

Perfectly or highly conserved DNA elements were found in vertebrates, invertebrates, and plants by various methods. However, little is known about such elements in protists. The evolutionary distance between apicomplexans can be very high, in particular, due to the positive selection pressure on them. This complicates the identification of highly conserved elements in alveolates, which is overcome by the proposed algorithm.

**Results:**

A novel algorithm is developed to identify highly conserved DNA elements. It is based on the identification of dense subgraphs in a specially built multipartite graph (whose parts correspond to genomes). Specifically, the algorithm does not rely on genome alignments, nor pre-identified perfectly conserved elements; instead, it performs a fast search for pairs of words (in different genomes) of maximum length with the difference below the specified edit distance. Such pair defines an edge whose weight equals the maximum (or total) length of words assigned to its ends. The graph composed of these edges is then compacted by merging some of its edges and vertices. The dense subgraphs are identified by a cellular automaton-like algorithm; each subgraph defines a cluster composed of similar inextensible words from different genomes. Almost all clusters are considered as predicted highly conserved elements. The algorithm is applied to the nuclear genomes of the superphylum Alveolata, and the corresponding phylogenetic tree is built and discussed.

**Conclusion:**

We proposed an algorithm for the identification of highly conserved elements. The multitude of identified elements was used to infer the phylogeny of Alveolata.

**Electronic supplementary material:**

The online version of this article (doi:10.1186/s12859-016-1257-5) contains supplementary material, which is available to authorized users.

## Background

### Introduction

*Ultraconserved elements* (UCEs) are perfectly conserved regions of genomes shared among evolutionary distant taxa. Usually it is assumed that these regions are identical in closely related species and have minor differences in relatively distant ones, which substantially limits the phylogenetic distances. Generally these are untranslated regions. UCEs were first described in mammals, where several hundreds of identical regions more than 200 bp in length have been reported [1]. Knockouts of certain UCE loci in mice resulted in viable fertile offspring suggesting a cryptic role of UCEs in genome biology [2].

Then, UCEs were found in other vertebrates as well as in invertebrates and plants. For example, hundreds of conserved noncoding sequences were detected in four dicotyledonous plant species: *Arabidopsis thaliana*, *Carica papaya*, *Populus trichocarpa*, and *Vitis vinifera* [3].

Makunin et al. [4] used preliminarily generated multiple alignments of insect genomes with the genome of *Drosophila melanogaster* as well as the genomes of vertebrates with the human genome; a 100-bp sliding window was used to find identical UCEs in drosophilids and vertebrates, and the number of UCEs in insects proved to be much lower compared to vertebrates. As an alternative, UCEs can be identified using the method described by Kent et al. [5] based on pairwise alignments of complete genomes generated by BLASTZ [6].

A number of studies such as [7–9] do not rely on genome alignments for the identification of UCEs, which seems generally advantageous. Thus, Reneker and Shyu [7] proposed an algorithm for the identification of short conserved DNA regions based on a hash table mapping all short words represented in the chromosome. It was used to search conserved sites and repetitive sequences in different prokaryotic and eukaryotic species. Later Reneker et al. [8] used this method with 8-bp words to identify and analyze UCEs of at least 100 bp in plants and to expand the UCE sets in vertebrates.

Christley et al. [9] offered a public domain software embedded into the BioCocoa library. It is based on a combinatorial algorithm that considers pairs of genomes using suffix arrays, after which the intersection is taken for all pairs; no alignments are involved. The method is claimed to be much faster than BLAST and is comparable in speed to the algorithms based on suffix trees. UCEs were identified in 17 genomes of vertebrates.

Identical UCEs are available in regularly updated databases and online resources such as UCbase [10] for humans and UCNEbase [11] for vertebrates from human to zebrafish.

UCEs can be used in phylogenetic studies. This relies on a phylogenetic signal that can be embedded in UCE flanks; their variation increases with the distance from the conserved core [12]. The conserved region allows easy alignment across widely divergent taxa, while variation in the flanks is useful for comparative analyses [13]. In this case, UCE-containing loci are sequenced in fairly closely related species. In particular, this method was applied to reconstruct the phylogeny of 15 avian species including *Pavo* and five co-distributed neotropical rainforest bird species [13, 14]. Similar studies were conducted on the phylogeny of birds in Neoaves [15], all major reptile lineages including tuatara [16], and fish [17]. McCormack et al. [18] used identical UCEs of reptiles (including birds) to design 2560 in silico probes, which were aligned with the available mammalian genomes, after which the flanks of the resulting regions were also aligned. The data obtained suggested the phylogeny of amniotes. Glazov et al. [19] have found all identical matches longer than 50 bp between the genomes of the mosquito *Anopheles gambiae* and two fruitflies *Drosophila melanogaster* and *D. pseudoobscura*, all of which belong to the order Diptera.

The average similarity between regions from UCE decreases as more distant taxa are included, which gave rise to the term *highly conserved elements* (HCEs). The identification of HCEs is also considered in numerous publications, many of which rely on alignments and percent identity-based methods [20–26].

After the identification of HCEs in placental mammals, the evolution was investigated by extending these HCEs to opossum, chicken, frog, and fugu [20]. The identified HCEs demonstrate minor differences in the corresponding regions, while their number substantially increased, to 13736. This work was further extended to elephant shark using the same approach [21].

The database Ancora provides non-exonic regions of high similarity between genome sequences from distantly related metazoan organisms: human, mouse, dog, horse, chicken, zebrafish, tetraodon, *Drosophila* (fruitfly), *Caenorhabditis* (roundworm), *Aspergillus oryzae* (fungus), and *Dictyostelium discoideum* (amoebazoan) [22]. These HCEs were identified by scanning pairwise whole-genome alignments obtained with BLASTZ for regions with identity of 70-100 % depending on the evolutionary distance.

Faircloth et al. [23] used pairwise alignments of the genomes of honeybee and *Nasonia vitripennis* to identify about 3000 of identical UCEs with the length of at least 40 bp, which were confined to HCEs with at least 80 % identity to their counterparts in two other insect genomes with a lower coverage (*Atta cephalotes* and *Solenopsis invicta*). Target sequencing of the selected HCEs in 30 species from different hymenopterans made it possible to refine the phylogeny of this taxon. The LASTZ program [24], an improved version of BLASTZ, was used in this analysis.

Siepel et al. [25] presented an approach to HCE identification based on multiple genome alignments. In this case, a comprehensive search was conducted separately in four groups of organisms: five vertebrate species (human, mouse, rat, chicken, and fugu), four insect species (three *Drosophila* species and *Anopheles gambiae*), two species of *Caenorhabditis*, and seven species of *Saccharomyces*. The elements were identified in multiple alignments using the phastCons program, which is based on a two-state phylogenetic hidden Markov model. Multiple alignments for each species group were prepared using MULTIZ, which builds a multiple alignment from local pairwise alignments of a designated reference genome with all other genomes in the group of interest [26], pairwise alignments were obtained by BLASTZ. As a result, from 68 thousand to 1.2 million HCEs with the length up to 1 kbp were predicted, and these HCEs are more phylogenetically justified and rich in secondary RNA structures.

Thus, ample data are available on the identification of both UCEs and HCEs for phylogenetic reconstructions of plants, fungi, and animals. However, little is known about UCEs and HCEs in protists.

### Summary

In contrast to the *special case* of identification of UCEs, sets of *identical* words in not too distant genomes, for which fast algorithms are available, the identification of HCEs, sets of notably different words in more evolutionary distant genomes, is a rather complex computational problem. To our knowledge, no fast algorithm is available for it. A number of studies performed HCE identification using pre-identified UCEs, which were supplemented by similar words from new genomes. Other studies relied on time-consuming generation of alignments rather than on pre-identified UCEs. In contrast to these approaches, the proposed algorithm identifies HCEs from scratch relying on neither known UCEs nor alignments, and it seems faster than other methods.

The standard methods based on the comparison of gene coding regions have been widely used to generate taxonomies and reconstruct species evolution over a long period of time. Later, the coding regions were supplemented by the primary and secondary structures of the regulatory regions of genomes (e.g., [27, 28]) as well as UCEs and HCEs. All these different approaches to taxonomy and evolution favor their better understanding. It bears repeating that HCE identification in distant species is a nontrivial computational problem. This particularly applies to apicomplexans due to different rates of their evolution as well as the rate of evolution in apicomplexans relative to that in better known animals and plants. Clearly, the HCE-based approach can only be used to supplement more traditional approaches to taxonomy and evolution. Comparisons of results obtained using HCE identification by the method proposed and those obtained using traditional methods are sampled below in the Results and Discussion (see The phylogeny of Alveolata). Specifically, the comparisons apply to the superphylum Alveolata and, in our next work, to mitochondria of ciliates (the phylum Ciliophora).

### Mathematical aspect

Mathematically, the identification of HCEs might be reduced to building and clustering a graph with nucleotide sequences assigned to its vertices. The edge weight usually reflects the similarity between the sequences at the edge ends. The weight is often computed from a global alignment using the Needleman–Wunsch algorithm or from a local alignment using BLAST.

The majority of clustering methods utilize various strategies to construct “heavily connected components,” i.e., the clusters that include only vertices mainly connected by high-weight edges. Various clustering approaches were proposed, from specifically organized partitioning of the spanning tree of the initial graph [27, 29] to time estimation of random walk on a graph (the OrthoMCL algorithm). In the latter algorithm based on Markov chains, a walk within a cluster should be long and jumps between clusters should be rare [30, 31]. The description of OrthoMCL implicitly states that its convergence is difficult to discuss even hypothetically. A single cluster can be found by the MEME algorithm [32], which seems to be well suited for general graphs not divided into parts.

Let us recall that a *multipartite* graph has a vertex partition into disjoint subsets (called *parts*). It does not have edges connecting vertices inside any part. A subgraph of the multipartite graph is called *m*-*dense* if each its vertex is connected to vertices from at least (*m*–1) different parts. Clearly, an *m*-clique (a clique of cardinality *m*) in a multipartite graph is an *m*-dense subgraph. Notice that there exist 3-dense 3-partite graphs that contain no 3-cliques. It is important to identify *m*-dense subgraphs with a high total weight that include not many vertices from the same part, e.g. subgraphs which contain no more than *γ* vertices from each part; the preferred *γ* value is 1 or very small. The *m*, *γ* and edge weight parameters should be given in advance. The identified *m*-dense subgraphs in a given multipartite graph can be screened one by one for cliques of cardinality *m* or less. Any *m*-dense subgraph with small value of parameter *γ* may be called a *cluster*. Worth mentioning is the clustering method that we used previously. When the size (cardinality) of a cluster is known in advance, e.g., in multicomponent systems where the length of the orthologous series is known for one component, the most dense cluster of this size is constructed using the algorithm described in [33, 34]. Closely related methods are described in [35–39].

Due to the heuristic nature of these procedures, the comparison of the algorithms is hard to formalize, especially in the absence of standard benchmarking data.

Here we propose a novel universally applicable and freely available software package for HCE identification [40]. Our software does not rely on known UCEs, nor HCEs, which makes it applicable to unstudied taxonomic groups. The package includes original algorithms for the graph generation and clustering, which allow the identification of HCEs that consist of not only similar regions but also relatively dissimilar regions from distant species. Specifically, the algorithms rely on the generation of a sparse multipartite graph (called the *source* graph) from pairs of similar regions, compaction of it into the so-called *initial* graph, and massively parallel identification of *dense* subgraphs in the latter graph, which are considered as HCEs.

### Species analyzed by the method

Many of apicomplexan species considered below are agents of protozoal infections: *Perkinsus marinus*, in mollusks; *Ichthyophthirius multifiliis*, in fish; many apicomplexans, in birds and mammals; and the full list of their hosts is much wider. The evolutionary distance between apicomplexans can be very high, in particular, due to the positive selection pressure on them; there are many relevant publications putting forward similar claims, e.g. [41]. This complicates the identification of highly conserved elements in alveolates, which is overcome by our algorithm.

Alveolates include four major lineages: ciliates, dinoflagellates, chromerids, and apicomplexans [42]. Chromerids are phylogenetically related to apicomplexan parasites and contain photosynthetic plastids, while apicomplexans have a non-photosynthetic plastid called the apicoplast [43, 44]. The coral-endosymbiotic algae *Chromera velia* and *Vitrella brassicaformis* share a common ancestry with apicomplexan parasites [45]. Plastids are also found in the dinoflagellates *Lepidodinium chlorophorum* [46], *Durinskia baltica*, and *Kryptoperidinium foliaceum* [47].

Neither *Cryptosporidium parvum* [48] nor *Gregarina niphandrodes* [49, 50] have plastids. Gregarines are early diverging apicomplexans. Thus, it is not clear whether the common ancestor of apicomplexans had plastids.

The protozoan parasite *Perkinsus marinus* is a facultative intracellular parasite of mollusks. Its close relationship with the Apicomplexa was initially proposed based on the ultrastructural analysis of the zoospore, which revealed the presence of organelles resembling an apical complex [51]. The transcriptome analysis of *P. marinus* suggests the presence of a relict plastid. Furthermore, *P. marinus* sequences display significant similarity to those from both apicomplexans and dinoflagellates [52].

*Tetrahymena thermophila* is a model ciliate species. It exhibits between non-coding RNA–genome interactions leading to the removal of one third of the genome in developing somatic nuclei [53]. Let us recall that the micronucleus and the macronucleus are separated inside the same cell, but they have a common genetic source. In vegetative life, the micronucleus is transcriptionally inert.

Finally, this work proposes a novel universally applicable and freely available software for HCE identification. Analysis of HCEs found with the help of it provided essential information on the phylogeny in the superphylum Alveolata.

## Methods

Following the Data subsection, we describe our major result, the method for highly conserved element identification.

### Data

All genomes analyzed are available either in the Eukaryotic Pathogen Database Resources [54] or in GenBank [55]. See Table 1 for details.

**Table 1:** All used species and their genome accession numbers

Organism	Source	Accession
Coccidia (apicomplexans)		
*Cyclospora cayetanensis strain CHN_HEN01*	GenBank	GCA_000769155.1
*Eimeria falciformis Bayer Haberkorn 1970*	EuPathDB	ToxoDB 26
*Hammondia hammondi strain H.H.34*	GenBank	GCA_000258005.2
*Neospora caninum Liverpool*	GenBank	GCA_000208865.2
*Sarcocystis neurona SN3*	GenBank	GCA_000727475.1
*Toxoplasma gondii ME49*	GenBank	GCA_000006565.2
Plasmodium (apicomplexans)		
*Plasmodium berghei ANKA*	EuPathDB	PlasmoDB 25
*Plasmodium chabaudi chabaudi*	GenBank	GCA_000003075.2
*Plasmodium falciparum 3D7*	GenBank	GCA_000002765.1
*Plasmodium yoelii yoelii YM*	EuPathDB	PlasmoDB 25
Piroplasmida (apicomplexans)		
*Babesia bovis strain T2Bo*	GenBank	GCA_000165395.1
*Babesia microti strain RI*	GenBank	GCA_000691945.1
*Theileria annulata strain Ankara*	GenBank	GCA_000003225.1
*Theileria equi strain WA*	GenBank	GCA_000342415.1
*Theileria orientalis strain Shintoku*	GenBank	GCA_000740895.1
*Theileria parva strain Muguga*	GenBank	GCA_000165365.1
Cryptosporidium (apicomplexans)		
*Cryptosporidium baileyi TAMU-09Q1*	EuPathDB	CryptoDB 26
*Cryptosporidium hominis TU502*	GenBank	GCA_000006425.2
*Cryptosporidium meleagridis UKMEL1*	EuPathDB	CryptoDB 26
*Cryptosporidium muris RN66*	GenBank	GCA_000006515.1
*Cryptosporidium parvum Iowa II*	GenBank	GCA_000165345.1
Other apicomplexans		
*Gregarina niphandrodes*	GenBank	GCA_000223845.4
*Ascogregarina taiwanensis*	GenBank	GCA_000172235.1
Chromerida (alveolata)		
*Chromera velia CCMP2878*	EuPathDB	CryptoDB 26
*Vitrella brassicaformis CCMP3155*	GenBank	GCA_001179505.1
Perkinsida (alveolata)		
*Perkinsus marinus ATCC 50983*	GenBank	GCA_000006405.1
Ciliophora (ciliates)		
*Tetrahymena thermophila SB210*	GenBank	GCA_000189635.1
*Paramecium tetraurelia strain d4-2*	GenBank	GCA_000165425.1
*Ichthyophthirius multifiliis strain G5*	GenBank	GCA_000220395.1
*Stylonychia lemnae 2x8/2*	GenBank	GCA_000325865.2

Unfortunately, the genome data contains many short contigs due to incomplete assembly. Nuclear chromosomes have been assembled for a few apicomplexan species: *Babesia bovis* [56], *Theileria parva* [57], *Theileria annulata*, *Neospora caninum*, *Toxoplasma gondii*, *Plasmodium* spp., and *Cryptosporidium* spp.

Also, we used genomes of *Ichthyophthirius multifiliis*, *Paramecium tetraurelia*, *Stylonychia lemnae*, and *Tetrahymena thermophila* from the phylum Ciliophora (ciliates) as well as *Perkinsus marinus* from the phylum Perkinsozoa.

### An overview of the method

Conceptually, the method performs a fast search for pairs of words (in different genomes) of maximum length with the difference below the specified edit distance. Such pair defines an edge whose weight equals the maximum (or total) length of words assigned to its ends. The graph composed of these edges is then compacted by merging some of its edges and vertices. The dense subgraphs are identified by a cellular automaton-like algorithm; each subgraph defines a cluster composed of similar inextensible words from different genomes. Almost all clusters are considered as predicted highly conserved elements.

**Fig. 1:**
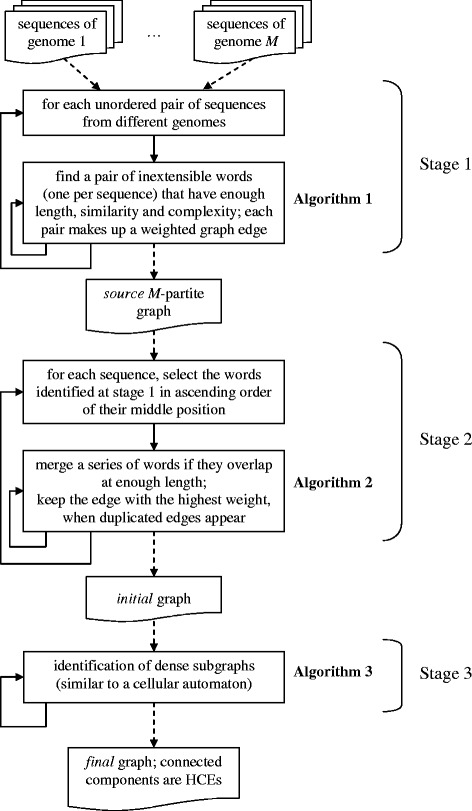
Stages and algorithms of the method

Technically, the method consists of the three following stages: a *source* graph is built from source data, this graph is used to construct an *initial* graph whose vertices and edges are generated by merging the vertices and edges of the source graph. The latter graph is used to construct the *final* graph containing only a fraction of vertices and edges of the initial graph. In the final graph the connected components are identified, which is the result of our method. These components are also called *clusters*. Sometimes, one of the clusters includes the great majority of vertices; such cluster is referred to as a *giant* cluster. A giant cluster is our method’s limitation; other clusters are considered as *predicted* HCEs. Figure 1 shows the main stages of the method.

An arbitrary region of sequence *A* between positions *i* and *j* (inclusive) will be referred to as a *word A*[*i*.. *j*]. The *edit distance λ* is defined for a pair of words [58, 59], which depends on fixed costs *δ*_*i*_, *δ*_*d*_, and *δ*_*s*_ of edit operations for character insertion, deletion, and substitution; they are subject to the usual constraints: all substitution costs are the same and *δ*_*i*_ = *δ*_*d*_ (the latter value will be referred to as *δ*_*id*_). The insertion/deletion cost is often set greater than that for substitution; below we assume all costs equal to 1 for simplicity. The edit distance equals the minimum number of operations transforming one word into another. Our software allows arbitrary costs; in this case the edit distance equals the minimum total cost of the series of operations. An arbitrary word *w* = 〈*w*_1_*w*_2_ … *w*_*l*_〉 *occurs* in sequence *A* = 〈*a*_1_*a*_2_ … *a*_*n*_〉, if there are positions 1 ≤ *i* ≤ *j* ≤ *n* in it, for which the edit distance *λ* (*w*, *A*[*i*.. *j*]) ≤ *ε*, where *ε* is a given threshold (parameter). This relationship will be referred to as *w* ≈ *A*[*i*.. *j*]. The threshold *ε* is usually not high; e.g., 6 character substitutions, insertions, and deletions for the word length *l* = 150*.*

Thus, we are given *M* genomes each of which is represented by a set of sequences using the same alphabet. Each set contains sequences with the total length not exceeding *N*. Good data correspond to well-assembled genomes (ideally, up to chromosomes or large scaffolds); in this case, the number of sequences in a set is relatively small. The *problem* is to find all words with length *l* ≪ *N* that occur in at least *m* ≤ *M* sequences from different genomes and to specify their positions in the sequences. Here *l* and *m* are parameters of the problem.

Let us make a remark on the algorithm memory and time complexities. If each genome consists of a single sequence *A* = 〈*a*_1_*a*_2_ … *a*_*N*_〉 of length *N*, the algorithm consumes the largest amount of memory, because it works with each pair of the top level sequences from each two genomes. The longer first sequence of the pair, the more memory is required. If the necessary amount of memory is not available, the sequence *A* may be split into parts with a small overlap. However, this requires the identified clusters to be analyzed for duplicated words occurring at the cuts. The time complexity depends on the total length of genomes irrespective of the number of sequences in those genomes.

### Parameters of the method

Let us list all parameters. Two of them reflect the dimension of the problem: the number of genomes *M* and the greatest total length of all sequences that belong to one of the genomes *N*. Next, there are two main parameters, the minimum allowable length of words to search *l* and the maximum edit distance between them ε. Since HCE characteristics are not known in advance, *l* and ε make it possible to control the trade-off between the prediction completeness and computation time. The value of ε is associated with the costs of replacement δ_*s*_ and insertion/deletion δ_*id*_ operations in our procedure of *semilocal alignment* (ref. to Additional file 1) of sequence regions; these three parameters are concordantly specified by the user based on the desired difference between words. The parameter *d* is the minimum length of overlapping between words in each group of words (see the Stage 2 section) that belong to the same sequence; this parameter modulates the integrity of clusters. Specifically, clusters are fragmented and merged as *d* increases and decreases, respectively. The values of *l* and ε uniquely determine the key length *k* and the maximum number of deletions *D* for lack of insertions in the same word, or vice versa (see note (2) in the Stage 1 section and Additional file 1). The values of *k* and *D* can be independently varied by the user to accelerate calculations, although it can sometimes cause underpredictions. The parameter *m* specifies the lower bound of the number of species (genomes) represented in HCEs; it is common to vary the *m* value. Accessory parameters include *t* (the maximum number of key copies in a sequence) and *r* (the maximum word compression ratio); these parameters can accelerate the algorithm by excluding low-complexity words from the search.

The main complication is the dimension of the problem to solve: the total sequence length *N* in each given set can be very high (the genome length of a typical protist is around 10^9^), and the number of sets *M* can be in the hundreds or more. The brute-force approach has a quadratic complexity with *O*(*M*^2^*N*^2^*l*^2^) comparison operations and the total memory of *O*(*MN*). This does not allow this problem to be solved even using current supercomputers. The known fast algorithms for the word search in a sequence (Boyer–Moore, Knuth–Morris–Pratt, etc.) are inapplicable here since they only find exactly matching words.

An efficient method based on our fast Algorithms 1–3 and tailored to parallel computation is presented below, in three stages (Fig. 1). These stages differ in scalability, so different number of processors or even distinct supercomputer can be appropriate for each of them.

### Stage 1: identification of candidate words in two sequences

The three following notes explain what underlies the high performance of our method.

(1) A straightforward quadratic algorithm checks the presence of each word from sequence *B* in the given sequence *A*. Instead of that we use indexing the sequence *A*, which is performed once and then reused many times.

(2) Highly conserved elements are identified so that the distance between words is low, i.e., the words become identical in no more than *ε* insertion, deletion, and substitution operations. Thus, target words a priori include *exactly* matching subwords of length *k* that can be used as search *keys*. For instance, the key length *k* ≈ 30 for the above parameters *ε* = 6 and *l* = 150.

(3) After matching keys are identified, each key in each of such pairs is extended in both directions as long as the distance between words (i.e., extended keys) does not exceed *ε*, their total length is the longest, and each word is not shorter than *l*.

The distance between words is usually evaluated using the Needleman–Wunsch global alignment algorithm [60] with the complexity of *O*(*l*^2^). But this algorithm assumes that the ends of words are fixed, while in our case only the beginning or end of a word is invariable depending on the extension direction. The application of local alignment algorithms (Smith–Waterman, BLAST, etc.) is excessive here since one of the word extremities is fixed (adjacent to the key extremity). Because of that, we use an original variant of the Needleman–Wunsch algorithm called *semilocal alignment*. Since this variant makes allowance for the threshold *ε* and uses a limit *D* of the number of deletions, its spatial and temporal complexity is *O*(*D l*), as shown in Additional file 1. Thus, semilocal alignment algorithm is linear in *l* as against the conventional quadratic algorithm.

**Fig. 2:**
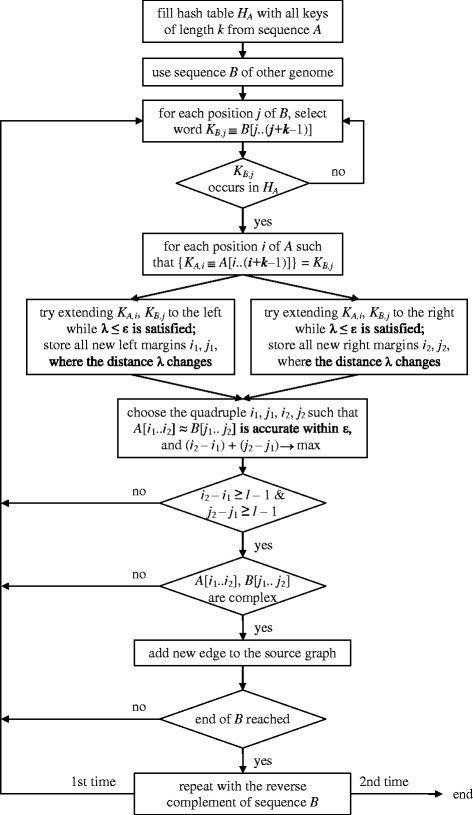
The flowchart of Algorithm 1

Based on the above three notes, the *Algorithm* 1 explained below is applied to each unordered pair of sequences 〈*A*, *B*〉 from different genomes (Fig. 2).

First, sequence *A* is indexed according to note (1) as follows. Let *k* be the key length set according to note (2) above. A hash table *H*_*A*_ is filled with all available keys, i.e., words of length *k* in sequence *A* along with their starting position in the sequence:

$$ {H}_A=\left\{{h}_A(i)\equiv \left({K}_{A,j},j\right)\kern0.5em \Big|\kern0.5em {K}_{A,j}=A\left[j..\left(j+k-1\right)\right],\kern0.5em i= Hash\left({K}_{A,j}\right)\right\}, $$

where *Hash*(⋅) is a hash function mapping the key to the table slot number. *Collisions* appearing as the hash table is generated decrease the algorithm efficiency. They stem from the imperfect hash function and duplications of the same word of length *k* in the source sequence. The rate of the former collisions can be reduced by selecting a different hash function and/or by decreasing the *load factor* of the table (the average number of keys per slot). The latter collisions cannot be avoided, although their rate decreases with *k*. Table 2 illustrates the rate of such collisions for real data (the complete genome of *Sarcocystis neurona, N* = 124377056). Here, such collisions at *k* = 16 appear in more than 20 % of cases; however, their rate decreases below 4 % as the key length increases to 24. Repetitive keys usually belong to genome regions of low complexity that are discarded here (see below); we use the occurrence threshold *t* for a key in the considered sequence, and keys that occur more than *t* times are discarded. Since the collisions are inevitable (although rare), we have chosen a hash table with separate chaining. The hash table size depends primarily on the length of sequence *A* and chosen load factor. Considering that sequence *B* is not involved here, the index is generated once for all sequences *A* in parallel and then repeatedly used for all *B* sequences in accordance with the note (1).

**Table 2:** The number of repeating keys of length *k* in the long sequence of the complete genome of *Sarcocystis neurona*

The number of occurrences of the key	*k = 16*	*k = 24*	*k = 32*	*k = 48*
1	94919216	119034327	121193592	121823700
2	5284353	764077	401812	242260
3, 4	1438861	190900	65945	24494
5–8	486137	60275	15503	4875
9–16	183799	20869	4129	1116
17–32	72581	7429	1202	194
33–64	29861	2996	437	155
65–128	11196	1217	208	85
129–256	4986	498	86	41
257–512	1776	148	9	3
513–1024	739	67	8	6
>1024	447	90	3	1
The number of different keys	102433952	120082826	121682934	122096930
The mean number of occurrences	1.21408	1.03559	1.02191	1.01833

After the hash table *H*_*A*_ is built, the following steps 1–6 of the Algorithm 1 are applied to one or more sequences *B*.

1. For each position *j* of sequence *B*, use the word *K*_*B*,*j*_ = *B*[*j*.. (*j* + *k* − 1)] as the key for the hash table.
2. Check if the key occurs in *H*_*A*_; if not, loop to step 1 using the next position *j*.
3. If there exists *s* such that *h*_*A*_(*s*) = (*K*_*B*,*j*_, *i*), a new pair of candidates has been found starting at positions *i* and *j* in sequences *A* and *B*, respectively. If the element *h*_*A*_(*s*) contains a chain of positions *j* due to multiple occurrences of the key, all (*i*, *j*) pairs are checked. However, we skip the pairs with a different key which can occur as a hash function limitation.
4. The identified pair of candidates is tested for key extension to approximately matching words of interest; if successful, the words are stored. The extension algorithm relies on the note (3); it is detailed in Additional file 1, where it has a complexity of *O*(*D l*).
5. Loop to step 1 with the next position *j* in sequence *B* until the end of the sequence.
6. Repeat steps 1–5 for the sequence reverse-complementary to sequence *B*.

If collisions are neglected, Algorithm 1 has a complexity of *O*(*N*) ⋅ *O*(*D l*), i.e., it is linear in the sequence length on average. The memory used, *O*(*N*), is also linear, although the corresponding constant can cause problems for very large *N*. In the case of a short alphabet, memory requirements can be reduced by compressing keys in the hash table *H*_*A*_: hashed keys *K*_*A*,*j*_ should be transcoded using a smaller number of bits per character and stored compressed in a smaller number of bytes; this does not affect key search in the table if candidate words are transcoded in the same way. Since the problem in question applies to sequences using a four-letter alphabet, memory requirements for storing keys in the hash table can be reduced fourfold; it is also convenient to select quadruple *k*.

To summarize, the overall complexity of the first stage of our method is *O*(*Nl*) for a single pair of sequences. For all unordered pairs, the complexity is multiplied by the number of them to become in the order of *O*(*M*^2^); however, sequence pairs can be processed in parallel as specified below.

Let us consider three more points relevant to Stage 1. Let us recall that HCE is considered as a set of words assigned to vertices of an *m*-dense subgraph (we temporarily neglect a possible giant cluster). We wish different HCEs to have the property designated as *inextensibility*: all words of one HCE are not subwords of words of another HCE taken from the same sequence locations. Naturally, the inextensibility requirement is limited to the set of all identified *m*-dense subgraphs. In order to meet this requirement, the key extension with the maximum total length of words is chosen at step 4 of Algorithm 1.

Only words of sufficient *complexity* are of interest. Complete genomes include long regions composed of one or two symbols repeated many thousand times, e.g., AT-repeats. HCEs containing words from such regions are ignored. The exclusion of simple highly repetitive words also decreases memory usage and accelerates our algorithm. This is realized also at step 4 by the Ziv–Lempel compression algorithm [61] implemented in the GNU gzip utility [62] with the threshold *r* empirically defined for the compression ratio.

In the presence of *M*(*M* − 1)/2 or more processors, Stage 1 is executed in parallel for each unordered pair of genomes independent of other pairs. The algorithm is most efficient when a sequence *A* of one genome is compared with all sequences *B* of other genomes in the separate process, in which case Algorithm 1 operates in parallel on *M* − 1 processors (although a greater number of processors can be beneficial considering that genomes are commonly represented by several sequences). Intermediate options with different numbers of parallel processes can be used to balance the computational burden between processors; we have considered this problem elsewhere [63].

The result of the first stage is a huge set of isolated edges; taken together they constitute the *source* graph with low vertex degrees. From our experience, the number of edges in it is typically closer to the number of vertices rather than to the squared number of vertices; the graph usually consists of many disjoint components. This is an edge-weighted graph; the edge *weight* is the maximum length of words assigned to its ends. The weight is evaluated at this stage and remains unaltered later. Sometimes it is more convenient to use a monotonic function of length of these words instead of their maximum length. A *graph part* is defined as all words of the source graph which belong to sequences of the same genome. There are no edges within a part. Thus, we get an *M*-partite graph that is further processed.

### Stage 2: compaction of the source graph

**Fig. 3:**
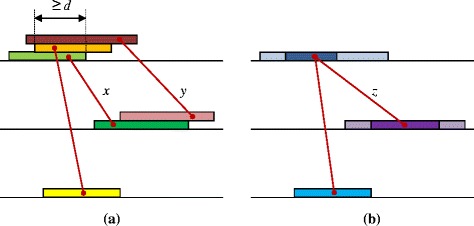
The compaction of the source graph by Algorithm 2: **a** three word pairs identified in three sequences and the corresponding edges of the source graph; **b** union of words at new vertices, the intersections are marked by a darker color; edges *x* and *y* merged into edge *z*

The properly extended keys are assigned to the ends of the edge defined by this pair of words in the source graph. Since edges are built at the first stage irrespective of the other edges, certain edges can have vertices corresponding to roughly the same location in a sequence but with different word ends, Fig. 3. At this stage, Algorithm 2 merges overlapping vertices along with incident edges, thus compacting the source graph. Specifically, Algorithm 2 checks words of each sequence in ascending order of their middle position. The maximum set (called *group*) of consecutive words from the same sequence that overlap by at least *d* characters forms a *new* vertex. If this requirement is not satisfied, the current word initiates a new group. Thus, each new vertex corresponds to its own group of consecutive words from the same sequence, and these groups are disjoint. This compaction of the source graph is done separately for each sequence, maybe in parallel.

During merging, old edges are first transferred to new vertices; however, if multiple edges emerge between two new vertices, the edge with the highest weight is kept. Each new vertex is assigned a *union* of all words of the corresponding group. The *intersection* of these words is also stored. Such unions and intersections are useful for the analysis of future HCEs. Each new vertex specifies an approximate position of an HCE word in the corresponding genome.

Thus, at the second stage the source graph is *compacted* by merging all vertices with corresponding words overlapping at a length of *d* or greater, thus producing so-called *initial* graph. The complexity of this stage is linear in the number of vertices in the initial graph and is naturally parallelized over the number of sequences.

### Stage 3: identification of dense subgraphs

At this stage, *Algorithm* 3 recognizes the *m*-dense subgraphs of the initial graph, primarily, with the highest total edge weight (Fig. 3). The set of identified *m*-dense subgraphs makes up the solution of the original problem. The resulting graph of this stage is referred to as *final*.

Algorithm 3 features deep internal parallelism and is similar in design to a cellular automaton with graph vertices as cells and incident vertices as neighboring cells. An independent process is initiated at step 1 shown below at each graph vertex. The processes at all vertices are synchronized after each step 1 or 2 so that a next step of any process starts only after the previous step was completed at all vertices.

**Fig. 4:**
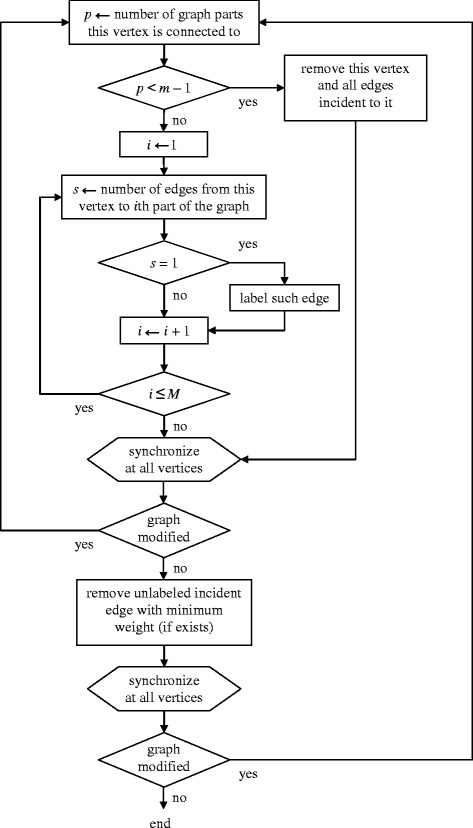
The flowchart of Algorithm 3

The two steps of Algorithm 3 are as follows (Fig. 4).

1. If a vertex is connected by edges to less than (*m* − 1) graph parts, this vertex and all edges incident to it are removed.

If a vertex is connected to a part by a single edge, such edge is labeled; labeled edges may be deleted only together with one of its ends.

If step 1 of the algorithm modified the graph, step 1 is executed again at all graph vertices.

Otherwise, step 2 is executed once at all graph vertices.

2. If an edge incident to the vertex is unlabeled and its weight is strictly less than those of all other unlabeled incident edges (or it is the only one), such edge is removed.

If step 2 modified the graph, step 1 is executed again at all vertices; otherwise the algorithm terminates. The result is the final graph. Each of its connected components is the desired *m*-dense subgraph.

The Algorithm 3 offers flexible scaling possibilities for the number of processors up to the number of graph vertices and can be implemented on distributed memory systems. If the number of available processors is less than the number of vertices, they are evenly distributed among the available processors, each of which executes the current step of the process at all allocated vertices one by one. The efficiency of parallel computation can be increased by allocating vertices connected by low-weight edges to the same processes and, vice versa, by allocating nonadjacent vertices or vertices connected by high-weight edges to different processes. The optimization of graph vertex allocation among processors is an independent problem. It is hard to evaluate the algorithm complexity analytically; however, it is not practicable since sample calculations of large data volumes demonstrated that the third stage of our method is executed much faster and requires a less powerful supercomputer than the first two ones.

A serious limitation of the method is the emergence of a connected component in the final graph including the majority of graph vertices when improper parameters are used to build the source and/or initial graphs; we referred to it as a *giant cluster*. The absence of such a component as well as the presence of *m*-dense subgraphs for *m* greater than the half of graph parts (i.e., the number of genomes) indicates a success. Our experiments demonstrated that the risk and size of the giant cluster can be decreased by removing all edges with the weight below a certain threshold, the choice of which is an independent problem. The presence of a giant cluster in the final graph not necessarily interferes with the identification of *m*-dense subgraphs with a smaller number of vertices for relatively high *m* values.

The substantial result of our method is a set of connected components of the final graph excluding the giant cluster, which are considered as predicted HCEs. Certainly, the definition of the giant cluster depends on the corresponding threshold.

Thus, we have developed a parallel algorithm that reduces the initial graph to the final graph composed of only *m*-dense subgraphs. Computer experiments on real data demonstrated that Algorithm 3 completes the task in a small number of such steps (usually numbering in the hundreds) even for very large graphs (e.g., 10^7^ vertices and 10^9^ edges, or even more). It also features flexible scalability for any number of available processors in a wide range.

## Results and Discussion

As mentioned above, our major result is the method for HCE identification set forth in the Methods section. The phylogeny of Alveolata section below discusses the results obtained by this method for biological data on the superphylum Alveolata.

All computing was made using the following parameters: *M* = 30, *N* = 1.97 ⋅ 10^8^, *l* = 65, *k* = 16, *t* = 200, *δ*_*s*_ = 1, *δ*_*id*_ = 2.1, *ε* = 17.5, *D* = 2, *r* = 2.2, *d* = 40, and *m* = 3.

### Comparison with LASTZ program

The first stage of our method has much the same goal as the pairwise alignment of genomes in many papers cited in the Background section. We used protists data to compare the results of our first stage with those of LASTZ with default parameters. The comparison was conducted in uniprocessor mode on a 2 GHz Linux workstation. Specifically, the longest (6.99 Mbp) chromosome of *Neospora caninum* was collated in turn with three well-assembled full genomes: *Babesia microti* of 4 chromosomes (6.39 Mbp in total), *Cryptosporidium parvum* of 8 chromosomes (9.1 Mbp), and *Plasmodium falciparum* of 16 chromosomes (23.3 Mbp). It took respectively 2 m 16 s, 11 m 35 s, and 30 h 47 m for LASTZ to process these three data sets. Our Algorithm 1 worked for 1 m 9 s, 1 m 30 s, and 20 m 26 s, respectively. It is arguable that our algorithm is faster and its complexity grows with input data volume not nearly as rapid as for LASTZ. The memory consumption was 130–204 MB for LASTZ and 620–645 MB for our algorithm. For the first data set, the number of pairs of similar words (aligned or identified) was 501 (LASTZ) and 108 (our algorithm). However, among of 501 alignments found by LASTZ, at least 202 were of low complexity regions; while our algorithm rejected such words. Remaining 299 alignments included less than a half (47 of 108) of word pairs found by our algorithm. This can be attributed to essentially different functionals used by the two approaches. Our algorithm looks for pairs of words that have the maximum length and the edit distance below the threshold, which results in words with the length of 61–109 bp (75 bp on average) and 72-85 % (77 % on average) of identity (similarity). LASTZ finds longer alignments (53–3117 bp; 273 bp on average), but with a lower identity (43-96 %; 66 % on average). The alignments identified by LASTZ included only 84 ones with the identity greater than 70 %, which is less than our algorithm has found. A similar situation is observed for two other data sets: LASTZ and our algorithm have found 36013 vs 7512 word pairs for the second set and 2.24 million vs 944 thousand word pairs for the third set, respectively.

The second and third stages of our method transit from a pairwise genome analysis towards building HCEs for a set of genomes. The authors are not aware of freely available counterparts of these tools. Generally speaking, they may not be necessary when HCEs are identified using a multiple alignment of genomes; each element becomes evident in many genomes [4]. In case of UCEs, the search using pairwise genome alignments can be based on intersections of pairwise elements [9]. In the general case, synthenic chains of elements in orthologous parts of genomes can be selected [5, 22].

### The phylogeny of Alveolata

**Fig. 5:**
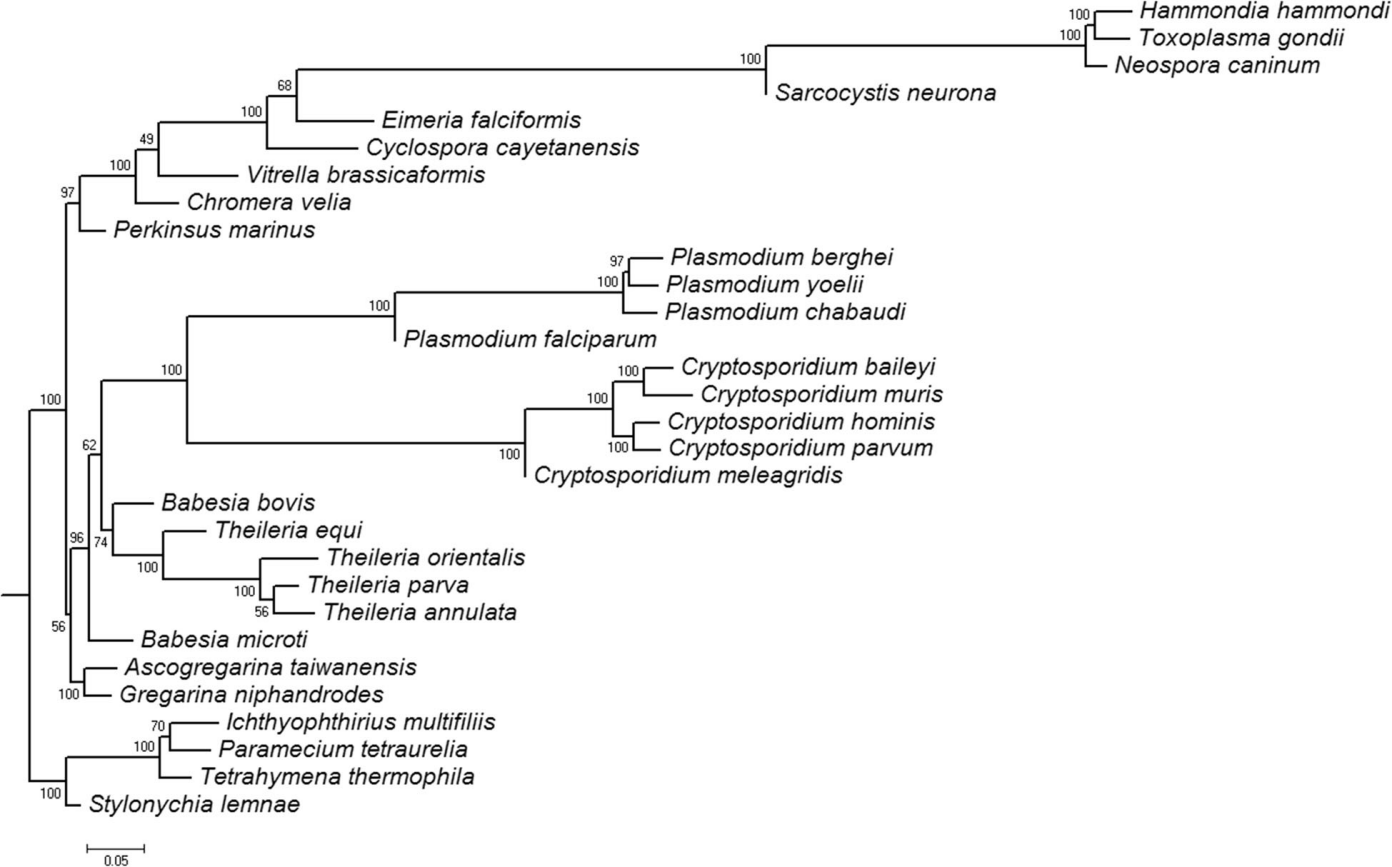
The tree predicted for 30 Alveolata species using their HCEs identified by our algorithm

The species tree predicted using the derived HCE data (Table 3) is shown in Fig. 5 and discussed below. The phylum Ciliophora was used as an outgroup to root the tree.

**Table 3:** Predicted HCEs

HCE type (label)	Count	Description
protein	8 988	A protein according to the GenBank annotation
tRNA	26	A transfer RNA
tRNA-Sec	1	Selenocysteine transfer RNA
LSU_rRNA	15	Large subunit ribosomal RNA
SSU_rRNA	5	Small subunit ribosomal RNA
5_8S_rRNA	1	5.8S ribosomal RNA
U1	1	U1 spliceosomal RNA
U2	1	U2 spliceosomal RNA
ACEA_U3	1	ACEA small nucleolar RNA U3
U4	1	U4 spliceosomal RNA
U5	1	U5 spliceosomal RNA
U6	3	U6 spliceosomal RNA
Protozoa_SRP	1	Protozoan signal recognition particle RNA (aka 7SL, 6S, 4.5S)
RNaseP_nuc	2	Nuclear ribonuclease P (RNase P)
snoR07	1	Small nucleolar RNA snoR07
snoR10	1	Small nucleolar RNA snoR10
SNORD36	1	Small nucleolar RNA SNORD36
intron	163	Non-coding region of a gene
unknown UCE	706	Not gene nor RNA predicted
Total	9919

Comparison of the complete genomes from Table 1 and generation of the source graph (Stage 1 of our method) were conducted on supercomputers MVS-100 K and MVS-10P in the Joint Supercomputer Center of the Russian Academy of Sciences [64], which required about 200 h on up to 512 processors. The subsequent compaction of the graph and identification of dense subgraphs (Stages 2 and 3 of the method) was conducted on a 32-core server with 256 GB RAM and required less than 20 h.

The source graph contained 32,028,631 vertices and 386,307,036 edges. Merging of properly overlapped vertices and removing duplicated edges yielded the initial graph with 3,867,747 vertices and 338,034,279 edges. After 185 steps of the algorithm for dense subgraph identification, the final graph included 2,153,534 vertices and 291,812,518 edges, which formed 9920 connected components. The giant cluster included 96.9 % vertices. It has not been analyzed in detail; however, some preliminary investigation demonstrated that many words in it *contain low complexity regions*. Complexity evaluation of individual regions in a word with overall allowable complexity is another problem not considered here. The remaining 9919 clusters included 67564 vertices. Detailed data on the obtained clusters including their words and summary data on clusters are available in Additional files 2 and 3, respectively.

All found words from HCEs were further analyzed using the following two resources.

1. Genome annotations available in GenBank (converted into GFF format); it was tested if a word of interest overlaps with the regions of a gene and its coding sequence (CDS). If both conditions are satisfied, the word corresponds to a protein (in this case, the data on both gene and its CDS are included in the Additional file 2); if only the first condition is satisfied, the word belongs to a gene untranslated region such as an intron (in this case, only the data on the gene is included in the Additional file 2).

2. Rfam database [65]; it was tested if the word is a fragment of a known non-protein-coding RNA. In this case, Additional file 2 specifies the RNA name and other data.

Additional file 3 summarizes the data on each cluster including the HCE type. Specifically, (1) if any of cluster words was found in Rfam, the cluster corresponds to a known RNA such as tRNA, snRNA, etc. and is labeled as this RNA; (2) otherwise if any of cluster words overlaps with a CDS, it corresponds to a protein (exon) and is labeled as “protein”; (3) otherwise if any of cluster words overlaps with a gene, it corresponds to an intron or other untranslated region and is labeled as “intron”; (4) otherwise the cluster describes an unknown HCE (no label).

Table 3 demonstrates that more than 90 % of identified HCEs correspond to proteins or known RNAs. Nevertheless, no data were found for 706 elements of unknown function as well as for 163 elements corresponding to non-coding gene regions. Although many of the latter genes are known or were reliably predicted, HCEs lie in their untranslated regions rather than exons (according to GenBank annotations). Many of such clusters include only a single word annotated as a presumable gene. These can be errors of automatic annotation. Anyway, these 163 HCEs deserve a thorough consideration together with those for which no data are available.

The above 163 clusters are largely small, i.e., the corresponding elements are conserved in a small number of species (12 at maximum and 3.08 on average). The number of clusters with 12, 6, 5, 4, and 3 species is 1, 2, 15, 28, and 823, respectively.

RAxML v. 8.2.4 [66] generated a tree (Fig. 5) using a matrix with 30 rows and 9919 columns corresponding to the number of nuclear genomes (species) specified in Table 1 and HCEs identified by our algorithm listed in Table 3. Cell value of 1 and 0 in the matrix indicates the presence or absence in a genome of an HCE-representing word, respectively. Branch lengths have standard meaning of estimated average number of substitutions (0 instead of 1 and vice versa) per site (between ancestral and descendant sequences). Thus, maximum likelihood search followed by rapid bootstrapping was performed in RAxML using binary substitution model with maximum likelihood estimate for the base frequencies. Frequency-based criterion was satisfied after 250 bootstrap replicates. All other RAxML parameters were left in default setting.

For all genera of apicomplexan parasites, their species group together in the tree. The only exception is *Babesia* spp. A separate position of *Babesia microti* relative to other species of the order Piroplasmida in the HCE-based tree is in agreement with its isolated position in the plastid tree [67]. At the same time, *B. orientalis* is closely related to other Piroplasmida species in agreement with [68].

The genus *Cryptosporidium* is not included in the class Coccidia in agreement with [69, 70]. It is closely related to the genus *Plasmodium*.

Two coral-endosymbiotic phototrophic algae *Chromera velia* and *Vitrella brassicaformis* are closely related, which agrees with [45]. Species from the subclass Coccidia have many common HCEs with phototrophic algae as well as with *Perkinsus marinus*. Here our result is in agreement with [52] as well as with an early observation by Levine [51].

Two species *Gregarina niphandrodes* and *Ascogregarina taiwanensis* are also closely related and compose an early diverged branch of the group of Apicomplexa.

According to our HCE-based tree, we can propose that the common ancestor of all the apicomplexans had no plastids. Thus, plastids could appear after the Gregarinasina isolation, while early apicomplexans with plastids were photosynthetic algae. Later these plastids were lost in *Cryptosporidium* spp.

Note a good agreement between the trees based on HCEs and all proteins encoded in the plastids of Apicomplexa and Chromerida species [71] as well as with chromosomal structures [72].

The isolated position of coccidians agrees with the results of comparative analysis of apicoplast-targeted proteins [73].

## Conclusions

We presented a novel algorithm to identify highly conserved DNA elements; it was applied to the superphylum Alveolata. The multitude of identified elements was used to infer the phylogeny of Alveolata which turned out to be in agreement with other available data. The described method for the identification of highly conserved elements is applicable to other fields where any texts are compared including natural language analysis targeted to identify the author, style, borrowings, etc. [74].

## Additional files

Additional file 1: Contains the details of step 4 of Algorithm 1 including auxiliary algorithms of semilocal sequence alignment and optimal key extension. (PDF 343 kb) Additional file 2: Presents in detail all clusters found in the final graph by our algorithm. The clusters are ordered by their numbers in column A; the first cluster is a giant one (shown partially). The first line of each cluster is marked with fixed numbers in columns C–D; it contains the number of vertices (words) of that cluster in column E. Each of the subsequent lines corresponds to a word and contains the following data in columns A–J: the cluster number (A), the number of species in the cluster (B), the vertex degree (C), the vertex density, i.e., the number of graph parts this vertex is connected to (D), the species name (E), the sequence name (F), start position of the word in the sequence (G), the word length (H), DNA strand indicator (I), and the word itself (J). A part of the word shown in capital letters corresponds to the intersection of all words merged at this vertex (a group); lowercase letters correspond to the union of those words. If the word overlaps with regions of a gene and its coding sequence (CDS) according to the genome annotation available in GenBank, this word corresponds to a protein. In such cases, the gene data including the protein description is shown in columns K–O; and CDS data, in columns P–R. If only the first condition is satisfied, the word belongs to a gene untranslated region such as an intron; in this case, only the data on the gene are shown. If a word is a fragment of a known non-protein-coding RNA according to Rfam database, columns S–AB contain the RNA name and other data. The clusters that correspond to untranslated regions or unknown HCEs are highlighted in gold or blue, respectively, in column A. (XLSX 10204 kb) Additional file 3: Presents summary data on the clusters. The Resume sheet provides some information on the algorithm results with different parameters. The variant with the threshold length 65 bp, which is discussed in the main paper, is highlighted in pink. In lines 15–42, the number of *m-*dense clusters and their vertices are shown for *m* values from 30 to 3. For example (line 15): 14 clusters were found containing words from all 30 genomes; these clusters comprise a total of 3736 vertices, i.e. 9 words per genome on average. Another example (line 35): 84 clusters were found containing words from 10 genomes; 1.2 words per genome on average. On the Clusters sheet, each line starting from the sixth one corresponds to a cluster. Column A contains the cluster number highlighted in the case of untranslated or unknown UCE (similar to Additional file 2). The HCE type is shown in column B as follows: if any of cluster words was found in Rfam, the cluster corresponds to a known RNA such as tRNA, snRNA, etc.; the column contains this RNA label; if any of cluster words overlaps with a CDS, it corresponds to a protein (exon) and is labeled as a protein; if any of cluster words overlaps with a gene, it corresponds to an intron or other untranslated region and is labeled as an intron; otherwise the cluster describes an unknown HCE (no label in column B). Column C shows the total number of words in the cluster; column D, the total number of species containing these words; and columns E–AH, the number of words from each species. (XLSX 1109 kb)

## Abbreviations

HCE: Highly conserved element
UCE: Ultraconserved element
