# Supplementary material for: A method for identification of highly conserved elements and evolutionary analysis of superphylum Alveolata
Source: BMC Bioinformatics. 2016 Sep 20;17:385. doi: 10.1186/s12859-016-1257-5 (PMC5028923; doi:10.1186/s12859-016-1257-5)
Supplement: Additional file 1: — Contains the details of step 4 of Algorithm 1 including auxiliary algorithms of semilocal sequence alignment and optimal key extension. (PDF 343 kb) [file 12859_2016_1257_MOESM1_ESM.pdf]

**Supplementary file 1: The extension of keys by original semilocal alignment**

Below are details of step 4 of Algorithm 1. We are given starting positions $i$ and $j$ of the same keys of length $k$ in sequences $A$ and $B$. We will individually extend the keys to a pair of *candidate words* meeting the following conditions: the distance between words does not exceed $\varepsilon$, the length of each word is not less than $l$, and they have maximum total length.

The distance between candidate words can be evaluated using a global alignment generated, e.g., by the Needleman–Wunsch algorithm; however, this approach is redundant since the keys will be aligned to each other in the case of typical length ratios (of keys and extensions). Accordingly, we will find independent alignments of the rightward and leftward extensions independently; one alignment boundary is given, it is adjacent to the key in each sequence. Here, *rightward* (*leftward*) extension is considered as an extension of both keys on the right (left). Such alignment will be referred to as a *semilocal* (right or left, respectively): one of its boundaries is fixed while the other is found by the algorithm. To make things simple, our procedure that extends the keys in one direction will also be referred to as a *semilocal alignment*. This procedure calculates the distance between current key extensions and implicitly defines the *ordering of position pairs* to which the keys were extended in a given direction in the sequences $A$ and $B$; the distance between current extensions cannot decrease.

Specifically, both keys are extended rightward until the distance between the current right extensions exceeds $\varepsilon$. List of position pairs is made for the right ends of extensions, for which the distance is strictly lower than that for any subsequent (according to our ordering) position pairs. Such list of pairs along with the distances between the corresponding extensions will be referred to as a *right* list. Similarly, both keys are extended leftward and the *left* list of position pairs is made for the left ends of extensions. The candidate word of the desired pair belongs to the region from the leftmost end of leftward extensions to the rightmost end of rightward extensions.

The result of this procedure, two candidate words, is schematically shown in Fig. 1 with specifically ordered pairs of positions ("*points*") in two given sequences $A$ and $B$ along the abscissa, and, along the ordinate, the distance between extensions that start from the corresponding key boundaries (beginning or end for leftward or rightward extension, respectively) and finish in a given point. Arrows indicate that the smaller of two values is taken in a plot breakpoint. The ticks on the horizontal axis denote pairs where the distance changes stepwise; these compose the lists mentioned above. Fig. 1 shows three position pairs in the left and right lists each; the lists can include the key boundaries.

Positions $[i, i+k-1]$ in sequence $A$ and $[j, j+k-1]$ in sequence $B$ belong to identical keys; and the distance between any corresponding parts of the keys equals zero. As the left or right boundaries of candidate words are shifted outward, the distance gradually increases but not necessarily immediately. Fig. 1 exemplifies a case where the sequences continued to match beyond the right key boundaries up to the ($i_4$, $j_4$) pair. After the left and right lists are complete, a pair of positions is selected in each of them so that the above conditions are satisfied. The ($i_1$, $j_1$) and ($i_2$, $j_2$) pairs are chosen in the left and right lists in Fig. 1, respectively. For instance, the selection of the ($i_0$, $j_0$) and ($i_3$, $j_3$) violates at least one condition: the total height in these points exceeds $\varepsilon$. Considering that $\varepsilon$ is low, the plot contains few steps and exhaustive enumeration of all variants causes no difficulties.

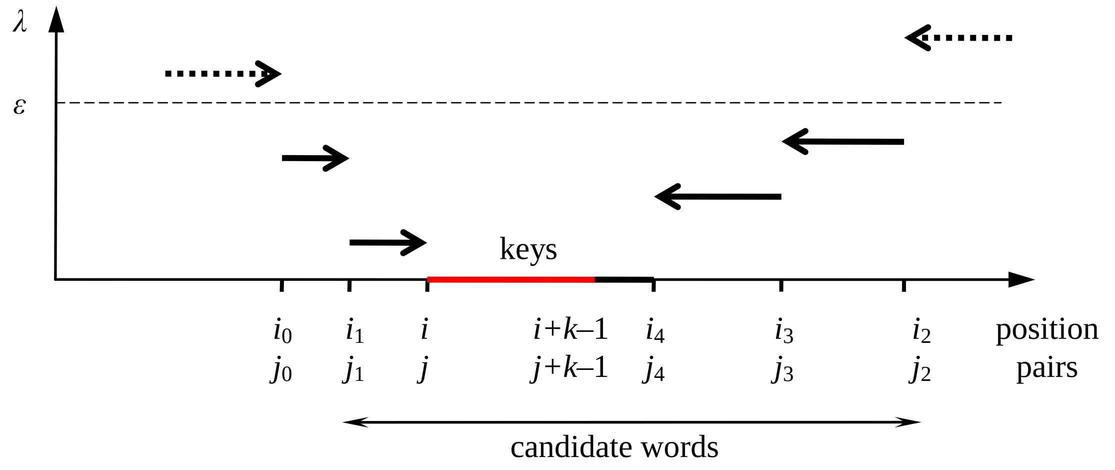


**Fig. 1.** The key extension method selects the specified variant of the desired candidate words in *A* and *B* (i.e., the pair of points) assuming that all other pairs of points define words that have strictly lower total length or the distance between them, equal to the total height at these points, exceeds $\varepsilon$. Two keys are shown in red.

Let us describe the proposed semilocal alignment algorithm for key extension in one direction, to be specific, towards the sequence ends (the rightward extension). Let us denote $a=\{a[p]: p=1,2,...,N_a\}$ and $b=\{b[q]: q=1,2,...,N_b\}$ parts of the sequences *A* and *B*, respectively, that are immediately adjacent to the key ends. The rightward extensions belong to these sequences and always start at position 1. The classical Needleman–Wunsch algorithm for the alignment of two sequences of lengths $N_a$ and $N_b$ is known to rely on the computation of two matrices *F* and *P* with the size of $(N_a+1)(N_b+1)$, which implies its quadratic spatial and temporal complexity. The matrix *F* stores the scores, and the matrix *P* stores the pointers, which are used for tracing back the path for the best alignment. At first, we used this algorithm despite its inefficiency in our case: positions *p*, *q* of sequences *a* and *b* to which the key is extended are not known in advance, while the matrices had to be fully computed irrespective of known threshold $\varepsilon$ for the maximum distance. This is why the following modifications were made (Fig. 2): only a region adjacent to the main diagonal and including *D* more neighboring diagonals upward and downward was considered in each matrix (Fig. 2, a). Essentially, the parameter *D* specifies the maximum allowed number of only insertions (if no deletions, or vice versa) in a sequence; the natural upper limit for it equals $\varepsilon/\delta_{id}$, although lower values can be adequate in practice. This region will be referred to as a *D-belt*. Recall that $\delta_{id}, \delta_s$ are respectively the costs of an indel and substitution operations.

Let us place selected *D*-belts of the matrices *F* and *P* in two matrices *G* and *Q* of the size of $(2D+1)\times(V+1)$, where $V \le \min\{N_a, N_b\}$ is an a priori unknown limit of the rightward extension (Fig. 2, b). The transition from coordinates *u*, *v* in the matrices *G* and *Q* to coordinates *p*, *q* in the matrices *F* and *P* (as well as sequences *a* and *b*) is performed using the equations $p=v+(u-D)^+$ and $q=v+(D-u)^+$, where $(\cdot)^+$ indicates the positive part, i.e. a quantity in parentheses if it is positive, and zero otherwise.

| | 0 | 1 | 2 | 3 | 4 | 5 | 6 | 7 | 8 | 9 | 10 |
|---|---|---|---|---|---|---|---|---|---|---|---|
| 0 | 2,0 | 1,0 | 0,0 | | | | | | | | |
| 1 | 3,0 | 2,1 | 1,1 | 0,1 | | | | | | | |
| 2 | 4,0 | 3,1 | 2,2 | 1,2 | 0,2 | | | | DIAG | UP | |
| 3 | | 4,1 | 3,2 | 2,3 | 1,3 | 0,3 | | | LEFT | | |
| 4 | | | 4,2 | 3,3 | 2,4 | 1,4 | 0,4 | | | | |
| 5 | | | | 4,3 | 3,4 | 2,5 | 1,5 | 0,5 | | | |
| 6 | | | | | 4,4 | 3,5 | 2,6 | 1,6 | 0,6 | | |
| 7 | | | | | | 4,5 | 3,6 | 2,7 | 1,7 | 0,7 | |
| 8 | | | | | | | 4,6 | 3,7 | 2,8 | 1,8 | 0,8 |
| 9 | | | | | | | | 4,7 | 3,8 | 2,9 | 1,9 |
| 10 | | | | | | | | | 4,8 | 3,9 | 2,10 |
| 11 | | | | | | | | | | 4,9 | 3,10 |

F, P

(a)

| | | | | | | | | | | | |
|---|---|---|---|---|---|---|---|---|---|---|---|
| 0 | 0,2 | 1,3 | 2,4 | 3,5 | 4,6 | 5,7 | 6,8 | 7,9 | 8,10 | | |
| 1 | 0,1 | 1,2 | 2,3 | 3,4 | 4,5 | 5,6 | 6,7 | 7,8 | 8,9 | 9,10 | |
| 2 | 0,0 | 1,1 | 2,2 | 3,3 | 4,4 | 5,5 | 6,6 | 7,7 | 8,8 | 9,9 | 10,10 |
| 3 | 1,0 | 2,1 | 3,2 | 4,3 | 5,4 | 6,5 | 7,6 | 8,7 | 9,8 | 10,9 | 11,10 |
| 4 | 2,0 | 3,1 | 4,2 | 5,3 | 6,4 | 7,5 | 8,6 | 9,7 | 10,8 | 11,9 | |

(b)

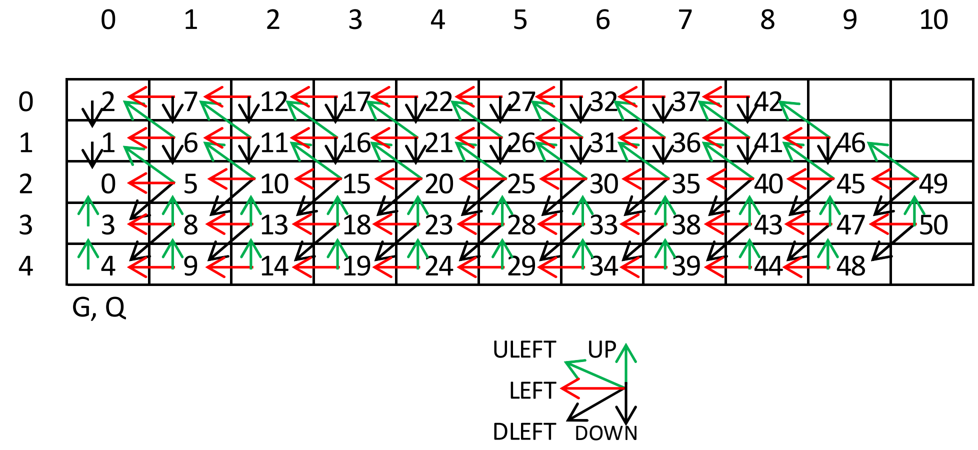


(c)

**Fig. 2.** New variant of Needleman–Wunsch algorithm: (*a*) initial matrix with *D*-belt for $N_a$=11, $N_b$=10, *D*=2; cells contain the positions of the elements in the transformed matrix; (*b*) position of *D*-belt in the transformed matrix; cells contain the initial positions of the elements; (*c*) the order of the transformed matrix elements computation and the directions of backtracking pointers.

In the transformed coordinates, the alignment algorithm operates in a conventional manner; however, the order of matrix element calculation and the direction of backtrack pointers should match those specified in Fig. 2, c. More specifically, the following pseudo-code is executed, where we use the notation

$$g(p,q)=\begin{cases}0 & \text{if } a[p]=b[q] \\ \delta_s & \text{if } a[p]\neq b[q]\end{cases}.$$

```
G[D][0] ← 0
for u=1 to D {
   G[D+u][0] ← u*δid
   G[D+u][0] ← u*δid
}
U ← 0
V ← 0
v ← 1
while (v ≤ Na and v ≤ Nb and G[U][V] ≤ ε) {
   Match ← G[D][v-1]+g(v,v)
   Delete ← G[D-1][v-1]+δid
   Insert ← G[D+1][v-1]+δid
   G[D][v] ← min(Match, Delete, Insert)
   if (min == Match)       Q[D][v] ← LEFT
   else if (min == Delete) Q[D][v] ← ULEFT
   else                    Q[D][v] ← DLEFT
   Best ← G[D][v]
   U ← D
   for u=1 to D {
      if (v+u ≤ Nb) {
         Match ← G[D-u][v-1]+g(v,v+u)
         if (u < D) Delete ← Big_number
         else       Delete ← G[D-u-1][v-1]+δid
         Insert ← G[D-u+1][v]+δid
         G[D-u][v] ← min(Match, Delete, Insert)
         if      (min == Match)  Q[D-u][v] ← LEFT
         else if (min == Delete) Q[D-u][v] ← ULEFT
         else                    Q[D-u][v] ← DOWN
         if (G[D-u][v] < Best) {
            Best ← G[D-u][v]
            U ← D-u
         }
      }
      if (v+u ≤ Na) {
         Match ← G[D+u][v-1]+g(v+u,v)
         Insert ← G[D+u-1][v]+δid
         if (u < D) Delete ← Big_number
         else       Delete ← G[D+u+1][v-1]+δid
         G[D+u][v] ← min(Match, Delete, Insert)
         if      (min == Match)  Q[D+u][v] ← LEFT
         else if (min == Delete) Q[D+u][v] ← UP
         else                    Q[D+u][v] ← DLEFT
         if (G[D+u][v] < Best) {
            Best ← G[D+u][v]
            U ← D+u
         }
      }
      V ← v
   }
}
```

The above pseudo-code corresponds to the forward run of the Needleman–Wunsch algorithm, which results in the edit distance of at most $\left(\varepsilon+\delta_s\right)$ in $G[U][V]$. Though the aligned extensions themselves are not required, the matrix of backtrack pointers $Q$ is still needed here to form the right list of end position pairs at which the distance between the words increases. It is done by tracing the pointers back to origin. Let us note that the backtracking path of the alignment algorithm must lie within the $D$-belt irrespective of the positions to which the keys are extended in a given direction; otherwise the limit $D$ of

deletions or insertions is violated. The backward run of the algorithm is presented by a pseudo-code below; it builds the right list (*Rlist*), which is the result of the keys extension to the right. Each entry $(i_r, j_r, \lambda)$ of this list is a triplet consisting of the right end positions of the extensions in sequences *A* and *B*, and the distance between them.

```
Last ← ε+δs
u ← U
v ← V
while (G[u][v] > 0) {
   if (G[u][v] < Last) {
      p ← v+i+k
      if (u > D) p ← p+u-D
      q ← v+j+k
      if (u < D) q ← q+D-u
      push (p,q,G[u][v]) → Rlist
      Last ← G[u][v]
   }
   else if (Q[u][v] == UP)
      u ← u-1
   else if (Q[u][v] == ULEFT) {
      u ← u-1
      v ← v-1
   }
   else if (Q[u][v] == LEFT)
      v ← v-1
   else if (Q[u][v] == DLEFT) {
      u ← u+1
      v ← v-1
   }
   else
      u ← u+1
}
```

The leftward key extension is performed in a similar way, which results in a left list. These left and right lists are used together to identify the optimal extension of the keys to candidate words in the two sequences as described above (see Fig. 1).

The spatial and temporal complexity of this algorithm equals $O(VD) = O(l)$ considering that $D \ll l$. Thus, the checking and extension of candidate words is performed using an algorithm *linear* in the length of words with the specified limit of the number of consecutive deletions.
